# Parsimony and Model-Based Analyses of Indels in Avian Nuclear Genes Reveal Congruent and Incongruent Phylogenetic Signals

**DOI:** 10.3390/biology2010419

**Published:** 2013-03-13

**Authors:** Tamaki Yuri, Rebecca T. Kimball, John Harshman, Rauri C. K. Bowie, Michael J. Braun, Jena L. Chojnowski, Kin-Lan Han, Shannon J. Hackett, Christopher J. Huddleston, William S. Moore, Sushma Reddy, Frederick H. Sheldon, David W. Steadman, Christopher C. Witt, Edward L. Braun

**Affiliations:** 1 Department of Biology, University of Florida, Gainesville, FL 32611, USA; E-Mails: tyuri@ou.edu (T.Y.); rkimball@ufl.edu (R.T.K.); kixs4@uga.edu (J.L.C.); hankin@ufl.edu (K.-L.H.); 2 Sam Noble Oklahoma Museum of Natural History, University of Oklahoma, Norman, OK 73072, USA; 3 4869 Pepperwood Way, San Jose, CA 95124, USA; E-Mail: jharshman@pacbell.net; 4 Museum of Vertebrate Zoology and Department of Integrative Biology, University of California, Berkeley, CA 94720, USA; E-Mail: bowie@berkeley.edu; 5 Department of Vertebrate Zoology, National Museum of Natural History, Smithsonian Institution, 4210 Silver Hill Road, Suitland, MD 20746, USA; E-Mails: braunm@si.edu (M.J.B.); huddlestonc@si.edu (C.J.H.); 6 Behavior, Ecology, Evolution and Systematics Program, University of Maryland, College Park, MD 20742, USA; 7 Zoology Department, Field Museum of Natural History, 1400 South Lakeshore Drive, Chicago, IL 60605, USA; E-Mail: shackett@fieldmuseum.org; 8 Department of Biological Sciences, Wayne State University, 5047 Gullen Mall, Detroit, MI 48202, USA; E-Mail: wmoore1415@gmail.com; 9 Biology Department, Loyola University Chicago, Chicago, IL 60660, USA; E-Mail: sreddy6@luc.edu; 10 Museum of Natural Science, 119 Foster Hall, Louisiana State University, Baton Rouge, LA 70803, USA; E-Mail: fsheld@lsu.edu; 11 Florida Museum of Natural History, University of Florida, Gainesville, FL 32611, USA; E-Mail: dws@flmnh.ufl.edu; 12 Department of Biology and Museum of Southwestern Biology, University of New Mexico, Albuquerque, NM 87131, USA; E-Mail: cwitt@unm.edu

**Keywords:** bird classification, avian phylogeny, nucleotide sequence alignment, total evidence, Columbiformes, Coraciiformes, Galliformes

## Abstract

Insertion/deletion (indel) mutations, which are represented by gaps in multiple sequence alignments, have been used to examine phylogenetic hypotheses for some time. However, most analyses combine gap data with the nucleotide sequences in which they are embedded, probably because most phylogenetic datasets include few gap characters. Here, we report analyses of 12,030 gap characters from an alignment of avian nuclear genes using maximum parsimony (MP) and a simple maximum likelihood (ML) framework. Both trees were similar, and they exhibited almost all of the strongly supported relationships in the nucleotide tree, although neither gap tree supported many relationships that have proven difficult to recover in previous studies. Moreover, independent lines of evidence typically corroborated the nucleotide topology instead of the gap topology when they disagreed, although the number of conflicting nodes with high bootstrap support was limited. Filtering to remove short indels did not substantially reduce homoplasy or reduce conflict. Combined analyses of nucleotides and gaps resulted in the nucleotide topology, but with increased support, suggesting that gap data may prove most useful when analyzed in combination with nucleotide substitutions.

## 1. Introduction

In DNA and protein sequence alignments, gaps are used to represent positions where insertion/deletion (indel) events have occurred, reflecting the absence of nucleotides or amino acids in specific sequences. Although indels accumulate in most genomic regions, they are more common in non-coding regions (e.g., introns) than in protein coding regions. Intron sequences have typically been used to examine relatively recent divergences (e.g., [1,2,3,4,5]), but there has been a growing appreciation that non-coding sequences also represent a rich source of phylogenetic information at deeper levels in vertebrate phylogeny. Indeed, non-coding data have been used to estimate phylogeny for a number of vertebrate orders (e.g., [6,7,8]) and classes (e.g., [9,10,11,12,13]).

The process of multiple sequence alignment results in the concurrent inference of gaps that reflect the position of indels [14]. Inferred gap positions are often coded as binary characters that reflect the hypothetical positions where insertions or deletions have occurred (hereafter, called “gap characters,” also see [9,15,16,17,18]), although more complex coding schemes are possible [19]. Regardless of the specific gap-coding scheme, including information about indels in phylogenetic analyses can increase the information available in multiple sequence alignments without requiring additional data collection [19,20]. In spite of this, few phylogenetic studies incorporate this information, usually treating gaps as missing data [21,22]. However, phylogenetic analyses that treat gaps as missing data can be statistically inconsistent, even when the model of sequence evolution is simple and the true alignment is available [22]. Moreover, the historical information available from gap characters may be especially valuable, since they appear to exhibit less homoplasy than nucleotide substitutions (e.g., [4,12,20]). Thus, identifying the best methods for coding and analyzing gap characters (or finding other approaches to incorporate indels into phylogenetic analyses) represents an important challenge.

Despite their potential value for phylogenetic analyses, gap characters also have the potential to be sources of error, just like other types of data. First, the multiple sequence alignment used to score the gap characters may be inaccurate. Alignment has a major impact upon phylogenetic estimation (e.g., [23,24,25,26,27]), even when gap characters are not analyzed. In fact, alignment error has been suggested to represent a fundamental problem for the use of non-coding regions to address deep divergences (e.g., [28,29]), although when examined carefully it is clear that phylogenetic analyses of some non-coding data matrices are relatively insensitive to the details of alignment (e.g., [30,31]). Finally, the indels that underlie gap characters may exhibit homoplasy. Some analyses of gap characters have reported misleading signal associated with gaps (e.g., [32,33]), including evidence for long-branch attraction [34]. These issues are expected to introduce error into analyses of gap character matrices, suggesting that empirical studies that establish the relative amounts of historical signal and noise associated with gaps scored for alignments of different types of sequence data.

The congruence of trees based upon gap characters and nucleotide substitutions for the same sequences can be used to assess performance of phylogenetic analyses of gap characters. Because gap characters typically exhibit less homoplasy than nucleotide substitutions (e.g., [20]), it is reasonable to hypothesize that gaps will have stronger phylogenetic signal than nucleotides. However, like other types of low homoplasy characters (e.g., [35]), changes in gap characters accumulate slowly, and this may limit their power to resolve difficult phylogenetic problems [36,37]. Most gap character matrices used in phylogenetic studies have been relatively small and, thus, have been unable to resolve phylogenetic relationships independently of nucleotide data. In fact, a recent study focused on avian phylogeny [38] included only 287 characters; analyses of those gaps alone were unable to resolve the avian tree. A few studies have used large numbers of gap characters [34,39], but those studies analyzed gaps in protein sequence alignments. Similar tests of the utility of gap characters from nucleotide sequence alignments of non-coding regions are desirable.

A rigorous test of the hypothesis that the phylogenetic signal in gap characters is stronger than that in nucleotides also requires a phylogenetic problem that includes at least some difficult to resolve nodes. The Hackett *et al*. [13] data matrix (hereafter, called the “Early Bird” data matrix) included nearly 4 million base pairs (bp) of avian sequence data, most of which were non-coding (74% intron and 3% UTRs). The number of gaps (12,030 characters) in this data matrix exceeds that in previous studies of non-coding regions by at least an order of magnitude, though Hackett *et al*. [13] did not consider gaps in their analyses. As avian phylogeny has been a difficult problem to resolve, analyses of a large-scale matrix of gap characters based on the Early Bird [13] data should provide an excellent test of the utility of gaps for phylogenetic analyses.

Here, we address five major questions about the utility of gap characters for phylogenetic analyses in avian non-coding regions. First, is the historical signal in the gap characters from Early Bird [13] stronger than, similar to or weaker than the signal in the nucleotide sequences? Second, do gap characters exhibit more or less homoplasy than nucleotides, and moreover, do gap characters based on the insertion or deletion of a single nucleotide exhibit more homoplasy than those based upon longer indels? Third, are the trees supported by gap and nucleotide characters congruent, and if not, which of the two trees is better corroborated by other lines of evidence? Fourth, does maximum parsimony (MP) or maximum likelihood (ML) represent a better method for analyses of gap characters, or do both methods perform similarly? Finally, are total evidence analyses that combine gap and nucleotide data superior to individual analyses of either data type? We expect the answers to these questions to provide insight into the phylogenetic utility of gap characters that are largely based upon indels in non-coding regions.

## 2. Methods

### 2.1. DNA Sequence Data, Alignment and Gap Coding

The Early Bird [13] data matrix comprises ~25 kilobases (kb) of sequence data per species (before alignment) from 19 nuclear loci obtained from 169 bird species (supporting information, file 1). The 19 loci are located on 15 different chromosomes in the chicken genome [40], and they are likely to be unlinked in most or all avian lineages given the general conservation of avian karyotypes [41]. There was clear evidence that one locus (*GH1*) underwent a gene duplication within birds [42]; a single *GH1* paralog was included for the taxa (Passeriformes) with two copies. Other details of the data matrix and alignment methods are provided in Hackett *et al*. [13] and Braun *et al*. [35].

The gap character matrix was generated using SeqState [43], which implements the simple indel coding method of Simmons and Ochoterena [19]. This method codes gaps as binary characters with “1” corresponding to presence of a gap (the absence of nucleotides) and “0” corresponding to absence of a gap (the presence of nucleotides). Gaps with different start and/or end positions are coded separately, and any gap that is enclosed within a longer gap is coded as missing (“?”) for taxa with the longer gap. Three gap matrices were generated, one based upon all indels, a second with gap characters based on indels longer than 1 bp and a third with gap characters based on indels longer than 2 bp. All data matrices are available from the Early Bird web site [44].

### 2.2. Phylogenetic Analyses

#### 2.2.1. Parsimony Analyses

We identified MP trees in PAUP* 4.0b10 [45] using the parsimony ratchet [46]. Ratchet searches reweight a random subset of characters and conduct searches using those perturbed matrices, permitting a more thorough exploration of treespace (for a detailed explanation see Nixon [46]). For this study, the ratchet analyses used 100 iterations with 20% of informative characters perturbed and one tree held per iteration. To conduct the ratchet analyses, we used a C++ program (written by E.L.B.) that generates an appropriate PAUP* block. After conducting 100 ratchet iterations, the optimal trees were retained and tree bisection, and reconnection (TBR) branch swapping was conducted to identify the full set of MP trees. When we compared this strategy to a more typical tree search (random additions of taxa followed by TBR branch swapping), we found that the ratchet took a shorter amount of time and identified shorter trees. Ratchet bootstrap analysis used 500 replicates, each of which used 100 ratchet iterations, as described above, with the final swapping limited instead to 1,000 trees per bootstrap replicate.

#### 2.2.2. Likelihood Analyses

Gap characters are binary, so a two-state Markov model (the Cavender-Farris-Neyman [CFN] model [47,48,49]) is appropriate for their analyses, at least in principle. However, all observed gap characters are by definition variable—their occurrence differs among taxa, otherwise they would not be discernible. Thus, gap characters exhibit an “acquisition bias” similar to that found in typical discrete morphological character matrices [50]. The acquisition bias for morphological data reflects the fact that most researchers only score parsimony informative characters; the failure to score uninformative characters is analogous to the inability to recover invariant gap characters (Felsenstein [51] referred to a similar phenomenon for restriction site data as “ascertainment bias”). Because of this issue, we employed a corrected CFN model that accommodates acquisition bias (we call this the CFNv model). The CFNv model is a special case of the more general Mkv model proposed by Lewis [50]; readers are referred to that publication for details. ML analyses using the CFNv model were conducted in PAUP* and GARLI v0.951 [52] after we converted the binary (01) gap characters to RY codes (0→R, 1→Y).

To correct acquisition bias in PAUP* and GARLI, we assumed that the observed variable characters (the gap matrix characters) were drawn from a larger, hypothetical data matrix with an unknown number of invariant characters. Then we approximated this hypothetical matrix by appending invariant characters (*i.e.*, columns that contain only “R” or “Y”) to the observed gap matrix. Then, the number of invariant characters necessary to maximize the conditional likelihood [50] of the resulting gap data was estimated by systematically adding invariant characters and calculating the likelihood in PAUP* using the CFN model with Γ-distributed rates (called the CF+Γ model in that program). A Java program written by T.Y. was used to automate the addition of equal numbers of all R and all Y columns. The impact of correcting for acquisition bias was evaluated by analyzing the data without the added sites, but most analyses were conducted using the optimal number of added invariant characters.

GARLI was used to search for the ML tree and to conduct likelihood bootstrap analyses. All analyses of gap data assumed equal state frequencies and a four-category discrete approximation to the Γ distribution (with the shape parameter estimated from the data). This corresponds to the CFNv+Γ model (for analyses with added invariant characters) or the CFN+Γ model (for analyses without added invariant characters). Up to 200 searches were conducted in GARLI to evaluate the ability of that program to identify the ML tree.

#### 2.2.3. Combined Analyses of Nucleotides and Gaps

We analyzed the gap data combined with invariant characters and nucleotide sequence data using PAUP* (for the MP criterion) and GARLI v0.96β (for the ML criterion). GARLI v0.96β is capable of analyzing partitioned data. The gap partition was analyzed using the CFNv+Γ model, as described above, whereas the nucleotide data were analyzed using the general time reversible (GTR) model with Γ-distributed rates and invariant sites (the GTR+I+Γ model). We estimated bootstrap support using 600 replicates.

### 2.3. Evaluating the Results of Phylogenetic Analyses Using Gap Characters

#### 2.3.1. Evaluating the Gap Phylogeny Using Congruence

**Figure 1:**
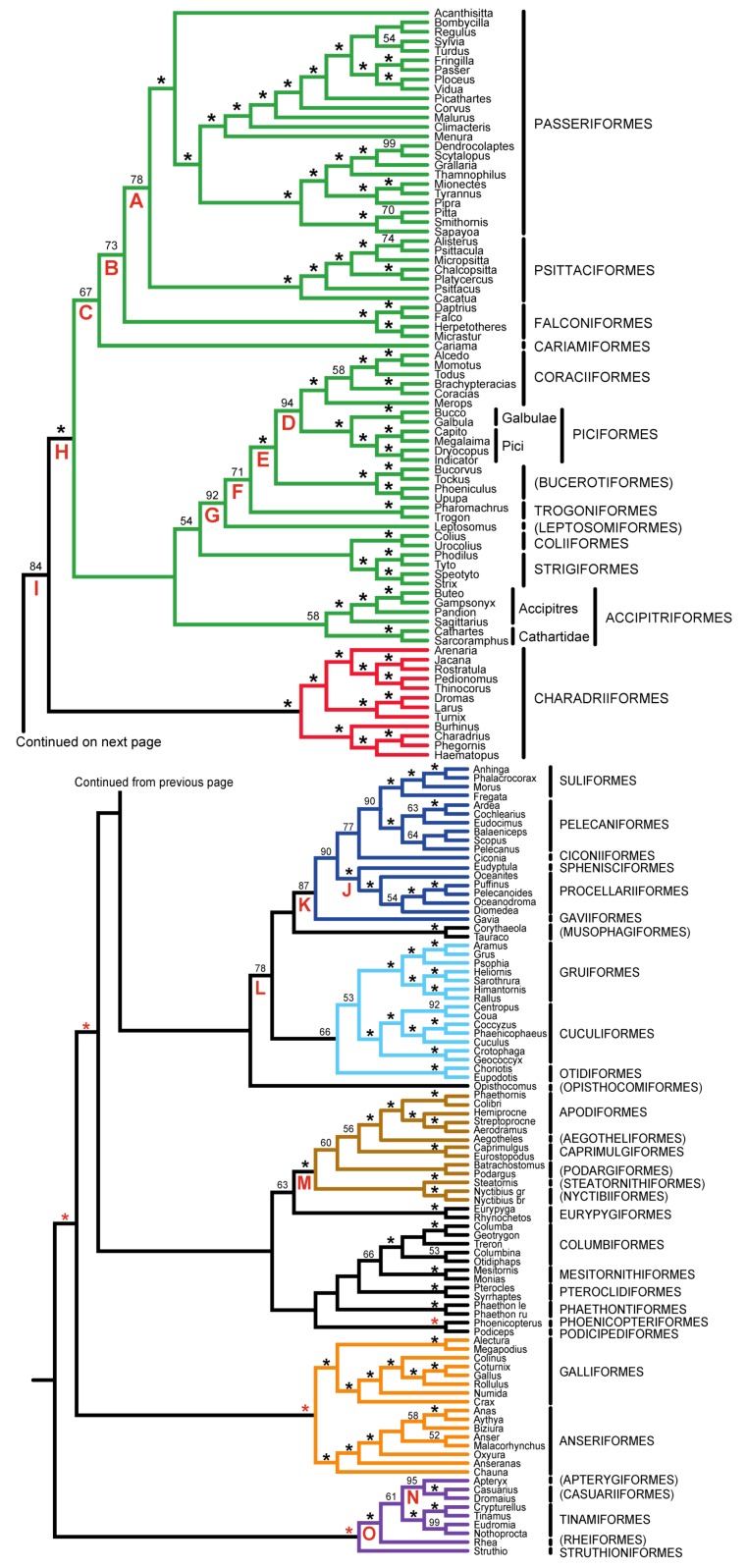
Estimate of avian phylogeny based upon nucleotide sequence data (maximum likelihood [ML] tree using the GTR+I+Γ model) and the higher-level classification described in the text. Nodes with 100% support are indicated with an asterisk. Red asterisks indicate nodes with 100% support that define supra-ordinal clades with extensive independent corroboration (see below). Coloring conventions here will be used in all trees, and named supra-ordinal clades are indicated using letters below branches (see Table 1 for details).

The best empirical method to assess the performance of novel phylogenetic methods or sources of phylogenetic information is to examine congruence with a known phylogeny [53] or, if such a phylogeny is unavailable, with topologies generated using independent data [30,54]. Unfortunately, most “known phylogenies” used to assess phylogenetic methods provide relatively weak tests, because they tend to include relatively easy to recover clades (see also Håstad and Björklund [55]). There are a number of strongly supported relationships in the avian tree of life (*i.e.*, those with 100% bootstrap support in Figure 1). These relationships were generally well supported and broadly accepted by avian systematists prior to the Early Bird study [56]; many of these clades correspond to orders in the Clements checklist [57] and the IOC World Bird List [58]. As these strongly supported relationships represent weak tests of phylogenetic methods, we will focus on the difficult to recover supra-ordinal clades present in the Early Bird tree.

Relationships among avian orders have proven to be very difficult to resolve and parts of the Early Bird tree may prove to be inaccurate. However, we note that a subset of the supra-ordinal clades present in the Early Bird tree have been corroborated to varying degrees by independent lines of evidence (Table 1). These independent lines of evidence include the results of analyses using mitochondrial genomes [28,59], transposable element (TE) insertions [60,61,62] and DNA hybridization [63] or phylogenetic analyses of nuclear gene regions not included in the Early Bird study [30,31,64]. Thus, these nodes represent difficult tests for phylogenetic methods, but they can nonetheless be viewed as “known” (with at least some degree of confidence) given their independent corroboration.

To facilitate discussion of the clades supported by the Early Bird tree, we have combined the classification used by Clements checklist [57] with a set of names for supra-ordinal clades (Table 1). The Clements classification was altered in two ways: non-monophyletic orders were split (in most cases, families were elevated to ordinal rank) and a broader circumscription (consistent with Wetmore [65] and the IOC World Bird List [58]) of Piciformes was used. In addition to facilitating the discussion of groups in this manuscript, we believe that the circumscriptions of ordinal and supra-ordinal clades that we present will be useful for two reasons: almost all orders are strongly supported by the bootstrap in the Early Bird tree, and the supra-ordinal clades can be mapped onto the commonly used checklists [57,58] in a straightforward manner.

The supra-ordinal clades listed in Table 1 are defined as the least inclusive clade comprising the relevant species in the Early Bird tree (see supporting information, file 1). Although the International Code of Zoological Nomenclature does not regulate names above the family level we have adhered to priority for several groups as much as possible (see references in Table 1). The name “Australavis”, the spelling published by Ericson [66], was modified to have the more appropriate ending “-es”. Priority for Strisores [67] is also somewhat problematic, so an alternative name (Cypselomorphae) proposed for a less inclusive clade, but sometimes used as a synonym, is also included in Table 1 (see supporting information, file 2, for additional details regarding the nomenclature of this group), but we retain that terminology. We have also proposed names for as yet unnamed clades; etymology for those names is provided in the supporting information (file 2).

Six strongly supported supra-ordinal clades were omitted from Table 1 (indicated with red asterisks in Figure 1). Four of these clades correspond to the major divisions in the avian tree of life: Palaeognathae (Struthioniformes and Notopalaeognathae), Galloanserae (or Galloanseres [68]; Galliformes and Anseriformes), Neoaves (all other extant birds) and Neognathae (Galloanserae and Neoaves). These clades have received extensive independent corroboration (reviewed by Cracraft *et al*. [69]). Daedalornithes (Aegotheliformes and Apodiformes [70]) and Mirandornithes (Podicipediformes and Phoenicopteriformes [71]) are also very strongly supported. These groups are typically recovered in phylogenetic trees based upon single genes (examples of individual gene analyses that support some or all of these groups include those based upon RAG1 [72,73,74], EGR1 [73] and up to 18 additional genes [13,75]). Thus, although these groups correspond to supra-ordinal clades, they do not represent difficult tests for phylogenetic methods.

**Table 1:** Supra-ordinal clades in the Early Bird tree and their corroboration by independent evidence (from mitogenomics [28,59], analyses of nuclear regions [30,31,63,64] that were not used by Hackett *et al*. [13] and transposable element (TE) insertions [60,61,62]). Strong corroboration (bootstrap support ≥70% or ≥3 TE insertions) was indicated using “++” and moderate corroboration (presence of the clade in with bootstrap support <70% or 1–2 TE insertions) was indicated using “+”. Blank cells indicate that the available independent evidence could not address the presence or absence of the clade, whereas “—” indicates evidence contradicting the clade. Citations for the introduction of clade names are included; names without citations were introduced here (supporting information, file 2).

		Support from Independent Evidence		
Clade	Name	Mitochondrial	Other Nuclear	TE insertions
A	Psittacopasserae [60]	—	+	++
B	Eufalconimorphae [60]	—	—	++
C	Australaves [66] (PPFC clade [30])	—	+	+
D	Picodynastornithes	—	++	
E	Picocoraciae [68]	—	++	
F	Eucavitaves (CPBT clade [30])	++	++	
G	Cavitaves			
H	Telluraves (“Landbirds” [13])	—	++	+
I	Litoritelluraves	—	+	+
J	Austrodyptornithes	+		
K	Aequornithes [68] (“Waterbirds” [13])	++		
L	Insolitaves	—	—	
M	Strisores [67] (Cypselomorphae)		+	
N	Novaeratitae	++	++	+
O	Notopalaeognathae	++	++	++

#### 2.3.2. Estimating the Rate at Which Gap Character Changes Accumulate

The rate of gap character change was estimated using ML estimates of branch lengths in the Early Bird [13] tree. Since branch lengths are expressed as substitutions per site (including invariant sites), estimates of branch lengths for gap characters include the added invariant characters. Thus, we multiplied the branch lengths based upon gap characters by the size of the gap character matrix (including the added invariant characters) and then divided by the size of the nucleotide matrix. This allowed the indel rate to be expressed as gap character changes per nucleotide site, making it directly comparable to nucleotide rates.

#### 2.3.3. Evaluating the Information Content of Gap Characters

The phylogenetic information content of gap characters relative to nucleotide data was evaluated using the ML bootstrap support of each node for trees estimated using each data type, but restricted to contain the same number of parsimony informative characters. When datasets differed in size, 100 jackknife pseudomatrices were generated; these reduced the number of parsimony informative sites in the larger dataset to that of the smaller. Each of these 100 jackknifed pseudomatrices was then bootstrapped, and the average bootstrap support values were used. We compared four pairs of data matrices: (1) all gap characters (4,245 informative characters) compared to gap characters based upon indels >1 bp in length (3,160 informative characters); (2) all gap characters compared to gap characters based upon indels >2 bp in length (2,640 informative characters); (3) all gap characters compared to nucleotide substitution characters; and (4) all gap characters compared to RY-coded nucleotide substitution characters (making the nucleotide data binary, like the gap characters).

## 3. Results and Discussion

### 3.1. The Power of Gap Characters to Resolve the Avian Tree of Life

**Figure 2:**
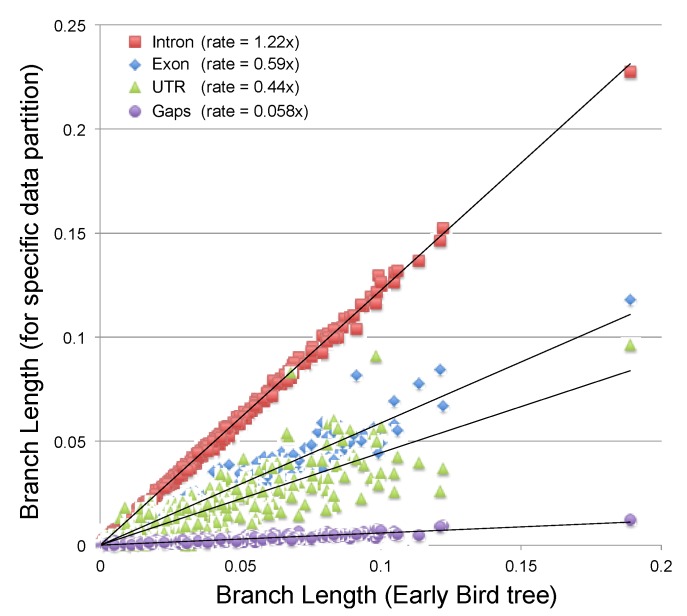
Branch lengths estimated from gap data (using the CFNv+Γ model) plotted against branch lengths from all nucleotide data (estimated using the GTR+I+Γ model). Branch length estimates for specific nucleotide partitions (introns, coding exons and 3' untranslated regions [UTRs]) are presented for comparison of relative rates (next page).

Resolving the topology deep in the avian tree of life is a notoriously difficult problem [12,76,77], making it an excellent test case for novel sources of phylogenetic information. The power of specific types of data to resolve phylogenetic relationships depends upon the size of the matrix, rate of evolution, amount of homoplasy and branch lengths in the true tree. The ideal evolutionary rate for phylogenetic characters is rapid enough for a high probability of synapomorphic changes to occur on the shortest branches in the tree, but not so high that homoplastic changes obscure historical signal [36,37]. The nucleotide substitution rate for introns appears to be appropriate for analyses of deep avian phylogeny [12]. In contrast, gap characters accumulate at a much lower rate (the MP treelength given gap data are approximately 10% of the treelength given nucleotide data). The ML estimate of the gap accumulation rate is even lower (Figure 2), although the lower homoplasy of gap characters may prove advantageous if very large gap datasets were analyzed.

**Figure 3:**
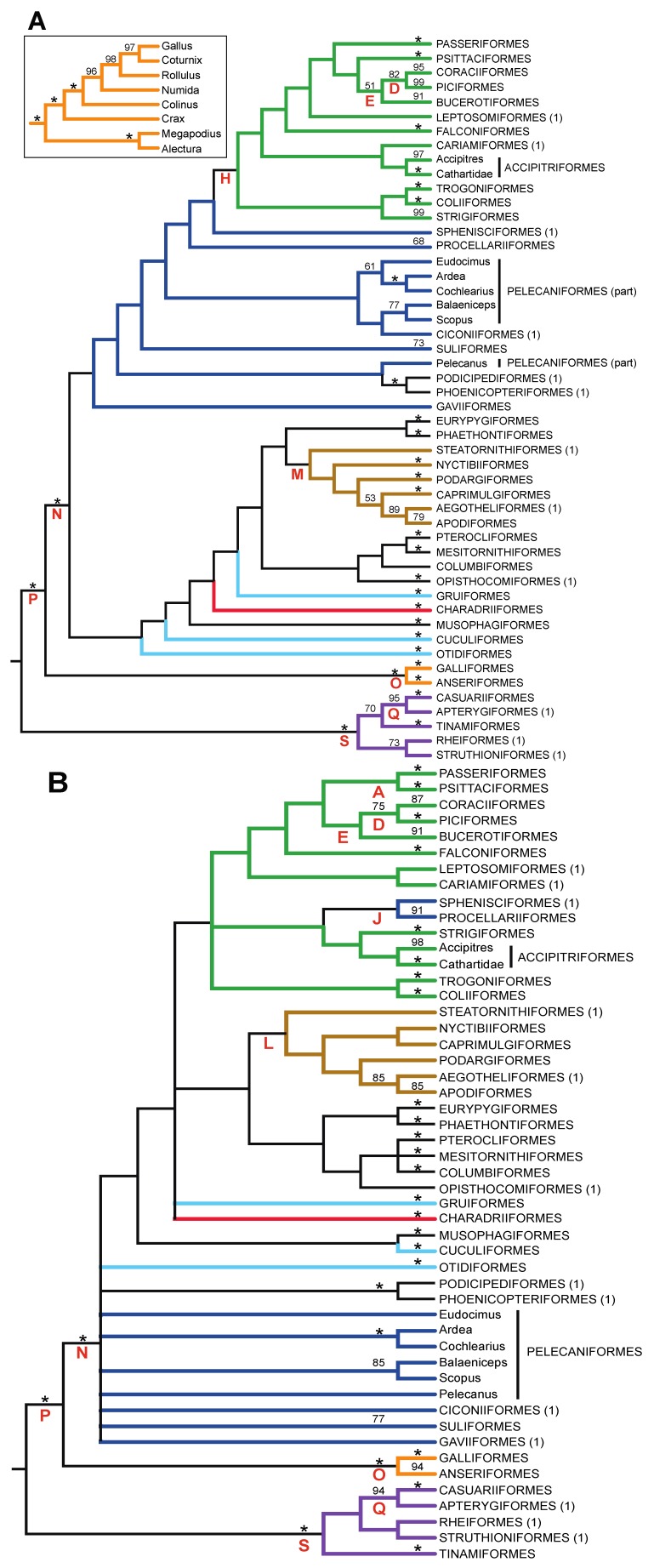
Estimates of avian phylogeny obtained using 12,030 gap characters obtained using (**a**) ML analyses with the CFNv+Γ model and (**b**) the maximum parsimony (MP) criterion. Orders were collapsed when monophyletic to simplify the trees. Bootstrap support on terminal branches reflects the support of those orders; orders represented by a single taxon are indicated using “(1)”. There were a limited number of rearrangements relative to the nucleotide topology within orders, most without bootstrap support. We highlighted the topology for the order Galliformes, because the gap topology included a clade with bootstrap support that conflicts with multiple nuclear gene regions [8,83] and morphology [84].

To examine the phylogenetic signal in gap characters, we obtained estimates of the avian tree of life based only upon gap characters (Figure 3 and supporting information, files 3 and 4). The gap tree had relatively high bootstrap support for most orders (Figure 3), the structure within orders (supporting information, files 3 and 4) and the small number of strongly supported supra-ordinal clades (*i.e.*, the clades indicated with red asterisks in Figure 1), albeit often with lower bootstrap support than the nucleotide tree. Those supra-ordinal groups recovered in the gap trees (e.g., Novaeratitae, Picocoraciae, Picodynastornithes and Strisores) were much more poorly supported by the bootstrap in the gap character tree than they were in the nucleotide tree. Other independently corroborated supra-ordinal clades were not even present in the gap tree (e.g., Telluraves). However, there was also an interesting exception; McCormack *et al*. [64] found a strongly supported Eurypygiformes-Phaethontiformes clade. This clade is present in the gap trees. We have refrained from suggesting a name for this clade, since it is absent from the Early Bird tree and lacks independent corroboration, but it could be a case where analyses of gap characters exhibit better agreement with other sources of information than the analyses nucleotides conducted by Hackett *et al*. [13]. Overall, these analyses demonstrated that a large gap character matrix has sufficient phylogenetic signal to recover many of the most strongly corroborated nodes in the avian tree of life, but few of the most difficult nodes.

**Figure 4:**
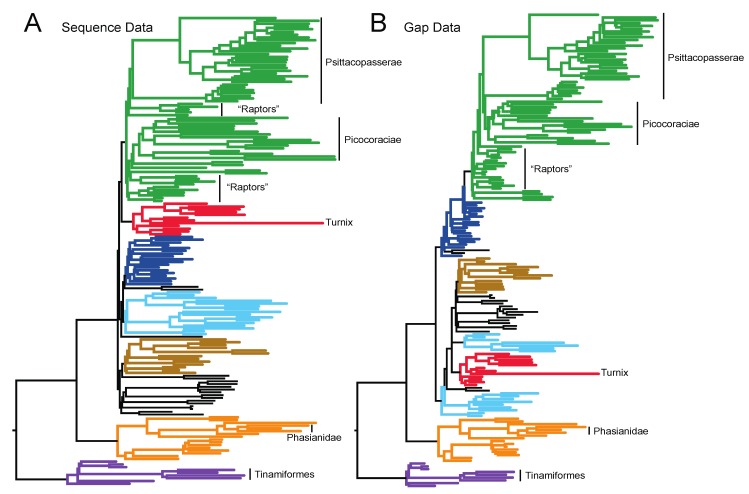
Branch length heterogeneity evident in the (**a**) optimal nucleotide tree (based upon the GTR+I+Γ model) and (**b**) the optimal gap tree (based upon the CFNv+Γ model).

Substantial branch length heterogeneity was evident in both the nucleotide and gap trees, and branch lengths appear to be somewhat correlated between the two data types (Figure 4). Several taxa have long branches relative to their close relatives, including *Turnix* (Charadriiformes), Tinamiformes (Paleognathae) and Phasianidae (represented here by the genera *Coturnix*, *Gallus* and *Rollulus* within the order Galliformes), in both the nucleotide and gap trees (Figure 4). This indicates that rates of nucleotide substitution and the accumulation of gap characters are correlated in birds, as expected based upon analyses of other groups of organisms (e.g., Hardison *et al*. [78]).

This branch length heterogeneity may influence the estimate of topology, and it is tempting to speculate that the clustering of the long-branched Psittacopasserae and Picocoraciae within Telluraves reflects long branch attraction, especially given the short branches associated with the raptorial taxa (Accipitriformes, Cariamiformes, Falconiformes and Strigiformes) within this supra-ordinal clade. If so, the gap tree would actually provide a less accurate estimate of avian phylogeny than the nucleotide tree given that both Psittacopasserae and Australaves are paraphyletic in the ML gap tree but strongly supported by independent evidence [30,60].

The observed branch length heterogeneity suggests that ML methods might provide better estimates of avian phylogeny than MP, because parsimony equivalent models (*i.e.*, the “no common mechanism” [NCM] model [79]) are unlikely to account effectively for branch length heterogeneity [80,81]. Indeed, it is clear that standard model selection approaches will indicate that the CFNv+Γ model has a better fit to the data than NCM [80], although we do note that there is debate regarding the question of whether MP should be viewed as a model [82]. Despite this prediction, our results are equivocal regarding the relative performance of these methods (e.g., compare Figure 3A to 3B). Indeed, the MP tree supports monophyly of Psittacopasserae and Austrodyptornithes (Figure 3B), unlike the ML tree, albeit with low (<50%) bootstrap support in both cases. The best interpretation of these differences between the MP and ML topologies is unclear, although differences between the trees at the supra-ordinal level provide no clear evidence that ML using the CFNv+Γ model performs substantially better than MP.

Examining other aspects of model fit, including conducting ML analyses without correcting for acquisition bias (*i.e.*, using the CFN+Γ model), also resulted in similar topologies. These equivocal results are most likely to reflect the limited phylogenetic information in gap data matrices, even ones as large as that analyzed here. This suggests that it will be necessary to examine even larger data matrices to determine whether either analytical approach provides an adequate fit to the underlying process of indel evolution and to establish the impact of these methods upon topology.

### 3.2. Phylogenetic Signal in Gap Characters Based upon Indels of Different Lengths

Above, we described two reasons why gap trees might have lower bootstrap support than the nucleotide tree. Specifically, the limited bootstrap support we observed could reflect the low rate of accumulation for gap character changes or poor model fit (alternatively, it could reflect a combination of both). Another possibility is that the gap data are sufficiently noisy that neither ML nor MP can recover an accurate estimate of the true tree. Even if noise is not positively misleading, it can have a negative impact upon the phylogenetic analyses [85]. Thus, noise reduction methods might provide a useful complement to model improvement. Short indels, especially 1-bp indels, are more common than long indels in avian non-coding regions [10,86], suggesting that gap characters based upon short indels may contain more noise than those based upon long indels. Thus, the removal of short indels has the potential to enhance phylogeny reconstruction.

To examine the utility of noise reduction based on gap length, we filtered the full gap data matrix (12,030 characters of which 4,245 were parsimony informative) and excluded gap characters based on short (1- and 2-bp) indels. Removing 1-bp gaps reduced the matrix size by almost 25% (to 9,115 characters; 3,160 parsimony informative), whereas excluding both 1- and 2-bp gap characters reduced the matrix size by an additional 11% relative to the original matrix size (to 7,740 characters; 2,640 parsimony informative). Although the rate of longer gap accumulation was lower (the rate after excluding 1-bp gaps is 76% of that for the all gap matrix and the rate after excluding 1- and 2-bp gaps is 64%) all three data matrices exhibit similar levels of homoplasy (Table 2). Estimates of phylogeny obtained after removing short indels did not improve congruence with the nucleotide data tree (supplementary information, file 2). Robinson-Foulds distances [87] between the nucleotide trees and all of the gap trees ranged from 92 to 100, whereas the distance among gap trees ranged from 64 to 70. Removing short gaps may prove beneficial for other data sets, but these results showed that 1- and 2-bp gaps did not contribute substantially to the noise in the gap dataset.

**Table 2:** Retention indices [88] for gap characters and nucleotide data. Retention indices were calculated using the ML topologies for nucleotides (Figure 1) or gaps (Figure 3a).

		Topology	
Data Matrix		Nucleotide tree	Gap tree
Gaps			
	All	0.7154	0.7209
	>1-bp (excluding 1-bp gaps)	0.7141	0.7190
	>2-bp (excluding 1- and 2-bp gaps)	0.7238	0.7288
Nucleotides			
	All	0.5231	0.5188
	Introns	0.5206	0.5167
	Coding exons	0.5315	0.5251
	3' untranslated regions	0.5632	0.5597

**Figure 5:**
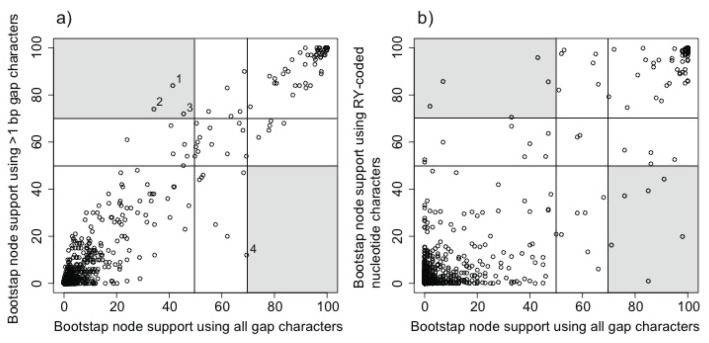
(**a**) Comparison of bootstrap support in trees based on all gap characters and gap characters >1-bp in length. Bipartitions that appeared well supported (≥70% bootstrap) by one analysis and poorly supported (<50% bootstrap) in the other are shaded. Numbers correspond to the following bipartitions: 1. *Ardea*-*Cochlearius-Eudocimus*; 2. *Alisterus*-*Psittacula*; 3. *Chalcopsitta*-*Platycercus*; and 4. Picodynastornites. (**b**) Comparison of bootstrap support for analyses using all gap characters and RY-coded nucleotide data. The same numbers of informative characters were used in each of these analyses (next page).

Not surprisingly, given the similar level of homoplasy in the full gap-data matrix and the filtered matrix with 1-bp gaps removed, bootstrap support in analyses using identical numbers of informative characters was similar in trees made from both data sets (Figure 5a). In fact, only four nodes exhibited fairly large changes in bootstrap support when 1-bp gaps were removed. In three cases, this was an improvement (from <50% to ≥70%); in the fourth case it was a decrease (from 69% to 12%). The node with reduced support united Picodynastornithes, a clade with independent corroboration (Table 1). In fact, Picodynastornithes was not present in the ML tree for gap data excluding 1-bp gaps; instead, the ML tree included a conflicting clade that comprised Coraciiformes and Bucerotiformes (supporting information, files 3 and 4). Similar results were obtained when both 1-bp and 2-bp gaps were excluded.

Surprisingly, the rearrangement within Picocoraciae observed when long gaps were excluded unites the “traditional” Coraciiformes. Morphological support for traditional Coraciiformes is mixed; traditional Coraciiformes form a clade in the analyses of Livezey and Zusi [89], whereas the analyses of Clarke *et al*. [90] conflict. However, we found it provocative that the gap trees support a *Momotus*-*Todus* clade, a topology that agrees with some morphological analyses [90,91] and conflicts with analyses of nucleotide data (Figure 1). However, none of the analyses of gap data had bootstrap support ≥70% for the *Momotus*-*Todus* clade.

In contrast to the modest differences between analyses using different gap data matrices, much larger differences were observed when we compared bootstrap support from the nucleotide and gap trees (Figure 5b). This observation does not reflect differences in state space because the nucleotide data were RY-coded to address the more limited character state space in the binary gap characters. These results suggest the existence of both congruent and incongruent signals in the gap and nucleotide data and indicate that the incongruent signals in the gap data were not disproportionately associated with gaps based upon the shortest indels.

### 3.3. Combined Analyses of Nucleotide Substitutions and Gap Characters

**Figure 6:**
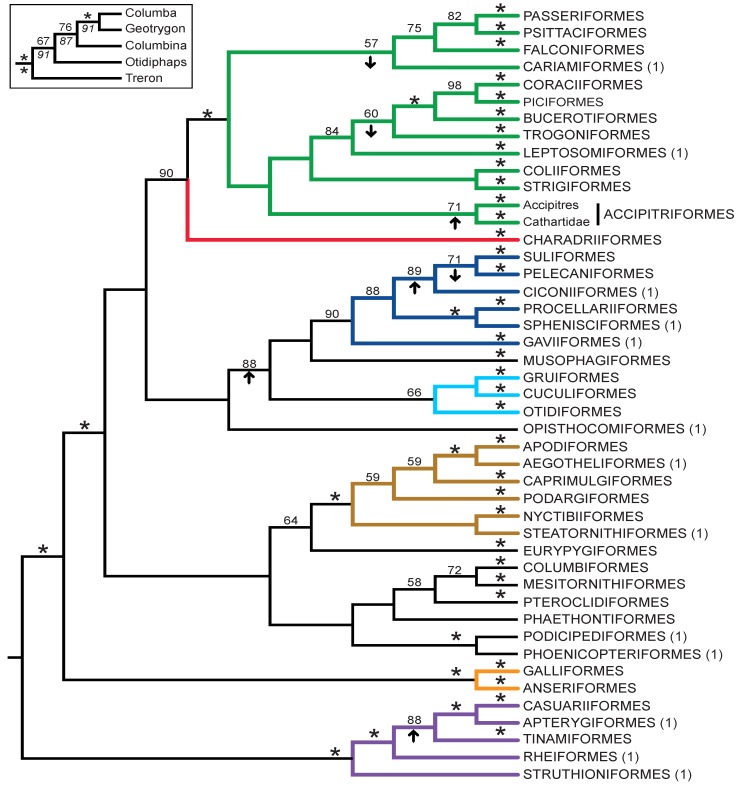
Combined evidence estimate of the avian tree of life. A partitioned ML analysis was conducted using the GTR+I+Γ model for the nucleotide partition and the CFNv+Γ model for the gap partition. Arrows indicate nodes defining supra-ordinal clades where bootstrap support increased or decreased by more than 10% relative to the nucleotide analysis (Figure 1). The combined evidence topology for Columbiformes was congruent with the gap topology instead of the nucleotide topology (inset; bootstrap values are reported for combined analysis [above branches] and for gap characters [below branches]).

ML analysis of the combined nucleotide and gap character data (including invariant gap characters) resulted in an estimate of phylogeny (Figure 6) virtually identical to the nucleotide tree (Figure 1). In general, there was a modest increase in the average bootstrap support for groups in the partitioned ML analyses of nucleotide substitutions and gap characters (Figure 6). However, there were also five nodes that exhibited more substantial increases in bootstrap support (>10%); four corresponded to supra-ordinal clades (Figure 6) and the fifth to the *Balaeniceps*-*Scopus* clade in Pelecaniformes (which increased to 75%). This general increase in support is consistent with the general assumption that including indel information in phylogenetic analyses would prove useful.

There were also four nodes in the combined evidence tree that exhibited fairly large (>10%) decreases in bootstrap support. These decreases were evident for three supra-ordinal groups (Figure 6) and the *Dendrocolaptes*-*Scytolopus* clade in Passeriformes (which decreased to 66%). There was another difference between the nucleotide and combined evidence trees within Columbiformes. The combined evidence topology for this order corresponded to that in the gap tree, where the relevant branches had even higher bootstrap support (Figure 6). Although the majority of differences between the nucleotide tree and the gap tree are likely to reflect the more limited power of gap characters to resolve phylogeny, these differences are likely to indicate the existence of conflicting phylogenetic signals in nucleotide substitutions and gap characters. These conflicts are likely to highlight nodes in the Early Bird tree [13] that should receive additional scrutiny.

There were two nodes with high bootstrap support in both the nucleotide (Figure 1) and gap trees (Figure 3) that conflicted; in both cases, the total evidence tree (Figure 6) was consistent with the nucleotide tree. Surprisingly, given the lower homoplasy of gap characters relative to nucleotide data (Table 2), independent evidence suggested that the nucleotide tree was more likely in both cases:

1. The nucleotide tree supports the monophyly of Notopalaeognathae in contrast to both the MP and ML gap trees (Figure 3), although only the latter had high bootstrap support. The nucleotide topology is strongly supported by independent evidence, including reanalyses of complete mitochondrial genomes [29], analyses of independent nuclear data matrices [31], TE insertions [62] and analyses of morphological data.
2. The nucleotide tree supports a clade comprising New World quail (*Colinus*) and Phasianidae within Galliformes (Figure 1), whereas the gap tree supports a clade comprising Guineafowl (*Numida*) and Phasianidae (Figure 3B). The former topology is supported by analyses of multiple nuclear and mitochondrial sequences [8,83], TE insertions [92] and morphology [84].

The combined evidence tree was virtually identical to the nucleotide tree, probably reflecting the ability of rapidly accumulating nucleotide changes to overwhelm the analysis. Nonetheless, the signal in gap characters appears to have an influence, because several supra-ordinal clades exhibited increases or decreases in bootstrap support ≥10% relative to the nucleotide tree. Support for the unnamed clade uniting Novaeratitae and Tinamiformes increased substantially. Although the existence of this clade is supported by analyses of complete mitochondrial genomes [29] analyses of independent nuclear data [31] were equivocal and two TE insertions [62] conflicted with the clade (there were no TE insertions consistent with the combined analysis). Likewise, support for Insolitaves also increased, although there is no independent evidence supporting this clade (Table 1). Finally, support for Accipitriformes, one the few orders with limited bootstrap support, also increased (from 58% to 71%). In contrast, two supra-ordinal clades with independent corroboration (Australaves and Eucavitaves) exhibited decreased support. Neither of those two clades appeared in the gap tree (Figure 3). The decreased support for Australaves and Eucavitaves in the combined evidence topology is consistent with the hypothesis that the nucleotide and gap data exhibit some genuine (albeit limited) conflict.

### 3.4. Analyses of Gap Characters and Models of Indel Evolution

The conflicts between the gap and nucleotide data may reflect the poor fit of the models we used for analysis. Better models of indel evolution are clearly desirable, because the actual patterns of indel evolution are no doubt more complex than the combination of gap coding and analyses using the CFNv+Γ model or parsimony-equivalent models. Indeed, it is unlikely that any of the models used in phylogenetics have a perfect fit to the underlying processes of sequence evolution. Nonetheless, approximating models have proven very useful for phylogenetic estimation (see Sullivan *et al*. [93] and Huelsenbeck *et al*. [81] for additional discussion). Thus, we felt that the simple ML approach we used represented a reasonable starting point that should be tested. However, we did not find this simple ML method performed substantially better than analyses using the MP criterion, suggesting future studies should explore more complex models.

Models of sequence evolution have improved along with our understanding of the processes of sequence evolution [81]. This raises the question of which aspects of indel evolution might prove to be most important for improving models of indel evolution. Although short indels are more common than long indels [10,86], we found that filtering the data matrix to remove short indels did not improve congruence, raising questions about the value of incorporating this correlation into models of indel evolution. The existence of a deletions bias has been established both for birds [17,86,94] and mammals [11], and incorporating this asymmetry might be useful. Indeed, asymmetry should be intrinsic to models of indel evolution; sequence alignments that represent evolutionary history accurately can include homoplastic deletions, but homoplastic insertions should be forbidden (since distinct insertion are, by definition, not homologous; e.g., Alekseyenko *et al*. [95]). Although more complex and realistic models that combine sequence and indel evolution in this manner have been proposed [95], it is unclear they can be implemented in a way that will prove to computationally tractable for phylogenies of this size. It also remains unclear whether these more complex models capture all of the relevant features of indel evolution, but the observation that analyses excluding indel information can be positively misleading [14,22] suggests that development of improved models of indel evolution remains critical.

Another aspect of model fit that should not be ignored is the assumption that a single tree underlies the observed distribution of gaps. Gene trees can differ from the species tree for several reasons [96]; for avian phylogeny, the most common reason is probably deep coalescence. The short branches at the base of Neoaves (Figure 4) suggest that incomplete lineage sorting due to deep coalescence was common during the radiation of this group [97]. The distribution of TE insertions is consistent with incomplete lineage sorting [60,61]. Discordance among gene trees is known to lead to the incorrect estimation of species trees when concatenated analyses are conducted [98], and we expect concatenated analyses of gaps from multiple loci to inherit all of the properties of similar analyses that use nucleotide data. Although nucleotide and gap data reflect the same genes and, therefore, the same set of gene trees, the number of gap characters and variable nucleotides differs among loci. These differences in the number of characters in each partition effectively result in differential weighting of loci in the gap and nucleotide trees and, therefore, create the potential for analyses of nucleotides and gaps to recover different topologies.

## 4. Conclusions

Our analyses indicated that a gap data matrix of more than 12,000 characters was unable to resolve the majority of difficult relationships in the avian tree of life (and, thus, were not clearly superior to nucleotide data), although the data did appear to improve bootstrap support when combined with nucleotide data. As expected, gaps accumulated much more slowly than nucleotide substitutions and this low rate likely limited their power for phylogenetic reconstruction. Rates of gap accumulation also differed among taxa in a manner correlated with the rate of nucleotide substitution. The observation that rates of gap accumulation differed among taxa suggested that model-based analyses (*i.e.*, ML with the CFNv+Γ model) might improve phylogenetic analyses of indels. However, we found only modest differences in performance between MP and ML. Additionally, removing short and potentially more homoplasious gaps did not improve tree reconstruction. Since the rate of gap character change is approximately an order of magnitude slower than the nucleotide substitution rate, it seems likely that at least an order of magnitude more data will be necessary to provide sufficient information to resolve the avian tree of life using indels alone. These larger indel datasets are likely to be available from birds very soon, and they should have the potential to contribute to the development of better models of indel evolution, improving future studies that include gap characters in many groups of organisms.

## Additional Note

While this manuscript was being reviewed Joel Cracraft generously provided a preprint describing the taxonomy used in the forthcoming *Howard and Moore Complete Checklist of the Birds of the World* [99]. I have included a brief description of the differences between the clade names used in that taxonomy and those used here in the supporting information (file 2).

## Supplementary Files

Supplementary File 1 Supplementary Information (ZIP, 104 KB)

## References

[1] DeBry R.W., Seshadri S. (2001). Nuclear intron sequences for phylogenetics of closely related mammals: An example using the phylogeny of *Mus*. J. Mammal..

[2] Kimball R.T., Braun E.L., Ligon J.D., Randi E., Lucchini V. (2001). A molecular phylogeny of the Peacock-pheasants (Galliformes: * Polyplectron* spp.) indicates loss and reduction of ornamental traits and display behaviors. Biol. J. Linn. Soc..

[3] Creer S., Malhotra A., Thorpe R.S., Pook C.E. (2005). Targeting optimal introns for phylogenetic analyses in non-model taxa: Experimental results in Asian pitvipers. Cladistics.

[4] Benavides E., Baum R., McClellan D., Sites J.W. (2007). Molecular phylogenetics of the lizard genus *Microlophus* (Squamata: Tropiduridae): Aligning and retrieving indel signal from nuclear introns. Syst. Biol..

[5] Igea J., Juste J., Castresana J. (2010). Novel intron markers to study the phylogeny of closely related mammalian species. BMC Evol. Biol..

[6] Harshman J., Huddleston C.J., Bollback J.P., Parsons T.J., Braun M.J. (2003). True and false gharials: A nuclear gene phylogeny of Crocodylia. Syst. Biol..

[7] Kimball R.T., Braun E.L. (2008). A multigene phylogeny of Galliformes supports a single origin of erectile ability in non-feathered facial traits. J. Avian Biol..

[8] Bonilla A.J., Braun E.L., Kimball R.T. (2010). Comparative molecular evolution and phylogenetic utility of 3'-UTRs and introns in Galliformes. Mol. Phylogenet. Evol..

[9] Prychitko T.M., Moore W.S. (2003). Alignment and phylogenetic analysis of β-Fibrinogen intron 7 sequences among avian orders reveal conserved regions within the intron. Mol. Biol. Evol..

[10] Fain M.G., Houde P. (2004). Parallel radiations in the primary clades of birds. Evolution.

[11] Matthee C.A., Eick G., Willows-Munro S., Montgelard C., Pardini A.T., Robinson T.J. (2007). Indel evolution of mammalian introns and the utility of non-coding nuclear markers in eutherian phylogenetics. Mol. Phylogenet. Evol..

[12] Chojnowski J.L., Kimball R.T., Braun E.L. (2008). Introns outperform exons in analyses of basal avian phylogeny using clathrin heavy chain genes. Gene.

[13] Hackett S.J., Kimball R.T., Reddy S., Bowie R.C.K., Braun E.L., Braun M.J., Chojnowski J.L., Cox W.A., Han K.-L., Harshman J. (2008). A phylogenomic study of birds reveals their evolutionary history. Science.

[14] Giribet G., Wheeler W.C. (1999). On gaps. Mol. Phylogenet. Evol..

[15] Kjer K.M., Gillespie J.J., Ober K.A. (2007). Opinions on multiple sequence alignment, and an empirical comparison of repeatability and accuracy between POY and structural alignment. Syst. Biol..

[16] Morrison D.A. (2009). Why would phylogeneticists ignore computerized sequence alignment?. Syst. Biol..

[17] Lee J.Y., Joseph L., Edwards S.V. (2012). A species tree for the Australo-Papuan Fairy-wrens and allies (Aves: Maluridae). Syst. Biol..

[18] Saurabh K., Holland B.R., Gibb G.C., Penny D. (2012). Gaps: An elusive source of phylogenetic information. Syst. Biol..

[19] Simmons M.P., Ochoterena H. (2000). Gaps as characters in sequence-based phylogenetic analyses. Syst. Biol..

[20] Simmons M.P., Ochoterena H., Carr T.G. (2001). Incorporation, relative homoplasy, and effect of gap characters in sequence-based phylogenetic analyses. Syst. Biol..

[21] Dwivedi B., Gadagkar S.R. (2009). Phylogenetic inference under varying proportions of indel-induced alignment gaps. BMC Evol. Biol..

[22] Warnow T. (2012). Standard maximum likelihood analyses of alignments with gaps can be statistically inconsistent. PLoS Curr..

[23] Lake J.A. (1991). The order of sequence alignment can bias the selection of tree topology. Mol. Biol. Evol..

[24] Ogden T.H., Rosenberg M.S. (2006). Multiple sequence alignment accuracy and phylogenetic inference. Syst. Biol..

[25] Smythe A.B., Sanderson M.J., Nadler S.A. (2006). Nematode small subunit phylogeny correlates with alignment parameters. Syst. Biol..

[26] Liu K., Raghavan S., Nelesen S., Linder C.R., Warnow T. (2009). Rapid and accurate large-scale coestimation of sequence alignments and phylogenetic trees. Science.

[27] Liu K., Linder C.R., Warnow T. (2010). Multiple sequence alignment: A major challenge to large-scale phylogenetics. PLoS Curr..

[28] Pratt R.C., Gibb G.C., Morgan-Richards M., Phillips M.J., Hendy M.D., Penny D. (2009). Toward resolving deep Neoaves phylogeny: Data, signal enhancement, and priors. Mol. Biol. Evol..

[29] Phillips M.J., Gibb G.C., Crimp E.A., Penny D. (2010). Tinamous and Moa flock together: Mitochondrial genome sequence analysis reveals independent losses of flight among ratites. Syst. Biol..

[30] Wang N., Braun E.L., Kimball R.T. (2012). Testing hypotheses about the sister group of the Passeriformes using an independent 30 locus dataset. Mol. Biol. Evol..

[31] Smith J.V., Braun E.L., Kimball R.T. (2013). Ratite non-monophyly: Independent evidence from 40 novel loci. Syst. Biol..

[32] Golenberg E.M., Clegg M.T., Durbin M.L., Doebley J., Ma D.P. (1993). Evolution of a noncoding region of the chloroplast genome. Mol. Phylogenet. Evol..

[33] Regier J.C., Zwick A. (2011). Sources of signal in 62 protein-coding nuclear genes for higher-level phylogenetics of arthropods. PLoS One.

[34] Belinky F., Cohen O., Huchon D. (2010). Large-scale parsimony analysis of metazoan indels in protein-coding genes. Mol. Biol. Evol..

[35] Braun E.L., Kimball R.T., Han K.-L., Iuhasz-Velez N.R., Bonilla A.J., Chojnowski J.L., Smith J.V., Bowie R.C.K., Braun M.J., Hackett S.J. (2011). Homoplastic microinversions and the avian tree of life. BMC Evol. Biol..

[36] Yang Z. (1998). On the best evolutionary rate for phylogenetic analysis. Syst. Biol..

[37] Braun E.L., Kimball R.T. (2001). Polytomies, the power of phylogenetic inference, and the stochastic nature of molecular evolution: A comment on Walsh *et al.* (1999). Evolution.

[38] Ericson P.G.P., Elzanowski A. (2011). Phylogenetic utility and evolution of indels: A study in neognathous birds. Mol. Phylogenet. Evol..

[39] Wolf Y.I., Rogozin I.B., Koonin E.V. (2004). Coelomata and not Ecdysozoa: Evidence from genome-wide phylogenetic analysis. Genome Res..

[40] Kimball R.T., Braun E.L., Bowie R.C.K., Braun M.J., Chojnowski J.L., Hackett S.J., Han K.-L., Harshman J., Heimer-Torres V., Holznagel W. (2009). A set of resources to amplify nuclear regions across the avian genome. Mol. Phylogenet. Evol..

[41] Shetty S., Griffin D.K., Graves J.A.M. (1999). Comparative painting reveals strong chromosome homology over 80 million years of bird evolution. Chromosome Res..

[42] Yuri T., Kimball R.T., Braun E.L., Braun M.J. (2008). Duplication and accelerated evolution of growth hormone gene in passerine birds. Mol. Biol. Evol..

[43] Müller K. (2005). SeqState: Primer design and sequence statistics for phylogenetic DNA datasets. Appl. Bioinformatics.

[44] Reddy S., Braun E.L. Assembling the Tree of Life: Early Bird. http://www.biology.ufl.edu/earlybird/.

[45] Swofford D.L. (2007). PAUP*. Phylogenetic Analysis Using Parsimony (*and other methods). Version 4.0b10.

[46] Nixon K.C. (1999). The Parsimony Ratchet, a new method for rapid parsimony analysis. Cladistics.

[47] Cavender J.A. (1978). Taxonomy with confidence. Math. Biosci..

[48] Farris J.S. (1973). Probability model for inferring evolutionary trees. Syst. Zool..

[49] Neyman J., Gupta S.S., Yackel J. (1971). Molecular studies of evolution: A source of novel statistical problems. Molecular Studies of Evolution: A Source of Novel Statistical Problems.

[50] Lewis P.O. (2001). A likelihood approach to estimating phylogeny from discrete morphological character data. Syst. Biol..

[51] Felsenstein J. (1992). Phylogenies from restriction sites: A maximum-likelihood approach. Evolution.

[52] Zwickl D.J. (2006). Genetic Algorithm Approaches for the Phylogenetic Analysis of Large Biological under the Maximum Likelihood Criterion.

[53] Russo C.A.M., Takezaki N., Nei M. (1996). Efficiencies of different genes and different tree-building methods in recovering a known vertebrate phylogeny. Mol. Biol. Evol..

[54] Miyamoto M.M., Fitch W.M. (1995). Testing species phylogenies and phylogenetic methods with congruence. Syst. Biol..

[55] Håstad O., Björklund M. (1998). Nucleotide substitution models and estimation of phylogeny. Mol. Biol. Evol..

[56] Harshman J., Jamieson B.G.M. (2007). Classification and phylogeny of birds. Reproductive Biology and Phylogeny of Birds.

[57] Clements J.F., Schulenberg T.S., Iliff M.J., Sullivan B.L., Wood C.L., Roberson D. The Clements Checklist of Birds of the World: Version 6.6. http://www.birds.cornell.edu/clementschecklist/downloadable-clements-checklist/.

[58] Gill F., Donsker D. IOC World Bird Names (v 3.2). http://www.worldbirdnames.org/.

[59] Pacheco M.A., Battistuzzi F.U., Lentino M., Aguilar R.F., Kumar S., Escalante A.A. (2011). Evolution of modern birds revealed by mitogenomics: Timing the radiation and origin of major orders. Mol. Biol. Evol..

[60] Suh A., Paus M., Kiefmann M., Churakov G., Franziska A.F., Brosius J., Kriegs J.O., Schmitz J. (2011). Mesozoic retroposons reveal parrots as the closest living relatives of passerine birds. Nat. Commun..

[61] Matzke A., Churakov G., Berkes P., Arms E.M., Kelsey D., Brosius J., Kriegs J.O., Schmitz J. (2012). Retroposon insertion patterns of neoavian birds: Strong evidence for an extensive incomplete lineage sorting era. Mol. Biol. Evol..

[62] Haddrath O., Baker A.J. (2012). Multiple nuclear genes and retroposons support vicariance and dispersal of the palaeognaths, and an Early Cretaceous origin of modern birds. Proc. R. Soc. B.

[63] Van Tuinen M., Butvill D.B., Kirsch J.A., Hedges S.B. (2001). Convergence and divergence in the evolution of aquatic birds. Proc. R. Soc. B.

[64] McCormack J.E., Harvey M.G., Faircloth B.C., Crawford N.G., Glenn T.C., Brumfield R.T. (2013). A phylogeny of birds based on over 1,500 loci collected by target enrichment and high-throughput sequencing. PLoS One.

[65] Wetmore A. (1960). A Classification for the Birds of the World.

[66] Ericson P.G.P. (2012). Evolution of terrestrial birds in three continents: Biogeography and parallel radiations. J. Biogeogr..

[67] Mayr G. (2010). Phylogenetic relationships of the paraphyletic “caprimulgiform” birds (nightjars and allies). J. Zool. Syst. Evol. Res..

[68] Mayr G. (2011). Metaves, Mirandornithes, Strisores and other novelties—A critical review of the higher-level phylogeny of neornithine birds. J. Zool. Syst. Evol. Res..

[69] Cracraft J., Barker F.K., Braun M., Harshman J., Dyke G.J., Feinstein J., Stanley S., Cibois A., Schikler P., Beresford P., Cracraft J., Donoghue M. (2004). Phylogenetic relationships among modern birds (Neornithes): Towards an avian tree of life. Assembling the Tree of Life.

[70] Sangster G. (2005). A name for the clade formed by owlet-nightjars, swifts and hummingbirds (Aves). Zootaxa.

[71] Sangster G. (2005). A name for the flamingo-grebe clade. Ibis.

[72] Groth J.G., Barrowclough G.F. (1999). Basal divergences in birds and the phylogenetic utility of the nuclear RAG-1 gene. Mol. Phylogenet. Evol..

[73] Chubb A.L. (2004). New nuclear evidence for the oldest divergence among neognath birds: The phylogenetic utility of ZENK (i). Mol. Phylogenet. Evol..

[74] Barrowclough G.F., Groth J.G., Mertz L.A. (2006). The RAG-1 exon in the avian order Caprimulgiformes: Phylogeny, heterozygosity, and base composition. Mol. Phylogenet. Evol..

[75] Ericson P.G.P., Anderson C.L., Britton T., Elzanowski A., Johansson U.S., Källersjö M., Ohlson J.I., Parsons T.J., Zuccon D., Mayr G. (2006). Diversification of Neoaves: Integration of molecular sequence data and fossils. Biol. Lett..

[76] Mindell D.P., Sorenson M.D., Huddleston C.J., Miranda H.C., Knight A., Sawchuk S.J., Yuri T., Mindell D.P. (1997). Phylogenetic relationships among and within select avian orders based on mitochondrial DNA. Avian Molecular Evolution and Systematics.

[77] Poe S., Chubb A.L. (2004). Birds in a bush: Five genes indicate explosive evolution of avian orders. Evolution.

[78] Hardison R.C., Roskin K.M., Yang S., Diekhans M., Kent W.J., Weber R., Elnitski L., Li J., O'Connor M., Kolbe D. (2003). Covariation in frequencies of substitution, deletion, transposition, and recombination during eutherian evolution. Genome Res..

[79] Tuffley C., Steel M. (1997). Links between maximum likelihood and maximum parsimony under a simple model of site substitution. Bull. Math. Biol..

[80] Holder M.T., Lewis P.O., Swofford D.L. (2010). The Akaike information criterion will not choose the no common mechanism model. Syst. Biol..

[81] Huelsenbeck J.P., Alfaro M.E., Suchard M.A. (2011). Biologically inspired phylogenetic models strongly outperform the No Common Mechanism model. Syst. Biol..

[82] Goloboff P.A. (2003). Parsimony, likelihood, and simplicity. Cladistics.

[83] Cox W.A., Kimball R.T., Braun E.L. (2007). Phylogenetic position of the New World quail (Odontophoridae): Eight nuclear loci and three mitochondrial regions contradict morphology and the Sibley-Ahlquist tapestry. Auk.

[84] Crowe T.M., Bowie R.C.K., Bloomer P., Mandiwana T.G., Hedderson T.A.J., Randi E., Pereira S.L., Wakeling J. (2006). Phylogenetics, biogeography and classification of, and character evolution in, gamebirds (Aves: Galliformes): Effects of character exclusion, data partitioning and missing data. Cladistics.

[85] Wenzel J.W., Siddall M.E. (1999). Noise. Cladistics.

[86] Han K.-L., Robbins M.B., Braun M.J. (2010). A multi-gene estimate of phylogeny in the nightjars and nighthawks (Caprimulgidae). Mol. Phylogenet. Evol..

[87] Robinson D.F., Foulds L.R. (1981). Comparison of phylogenetic trees. Math. Biosci..

[88] Farris J.S. (1989). The retention index and the rescaled consistency index. Cladistics.

[89] Livezey B.C., Zusi R.L. (2007). Higher-order phylogeny of modern birds (Theropoda, Aves: Neornithes) based on comparative anatomy: II.—Analysis and discussion. Zool. J. Linn. Soc..

[90] Clarke J.A., Ksepka D.T., Smith N.A., Norell M.A. (2009). Combined phylogenetic analysis of a new North American fossil species confirms widespread Eocene distribution for stem rollers (Aves, Coracii). Zool. J. Linn. Soc..

[91] Mayr G., Mourer-Chauviré C., Weidig I. (2004). Osteology and systematic position of the Eocene Primobucconidae (Aves, Coraciiformes *sensu stricto*), with first records from Europe. J. Syst. Paleontol..

[92] Kriegs J.O., Matzke A., Churakov G., Kuritzin A., Mayr G., Brosius J., Schmitz J. (2007). Waves of genomic hitchhikers shed light on the evolution of gamebirds (Aves: Galliformes). BMC Evol. Biol..

[93] Sullivan J., Swofford D.L. (2001). Should we use model-based methods for phylogenetic inference when we know assumptions about among-site rate variation and nucleotide substitution pattern are violated?. Syst. Biol..

[94] Johnson K.P. (2004). Deletion bias in avian introns over evolutionary timescales. Mol. Biol. Evol..

[95] Alekseyenko A.V., Lee C.J., Suchard M.A. (2008). Wagner and Dollo: A stochastic duet by composing two parsimonious solos. Syst. Biol..

[96] Maddison W.P. (1997). Gene trees in species trees. Syst. Biol..

[97] Oliver J.C. (2013). Microevolutionary processes generate phylogenomic discordance at ancient divergences. Evolution.

[98] Edwards S.V. (2009). Is a new and general theory of molecular systematics emerging?. Evolution.

[99] Cracraft J., Dickinson E.C., Remsen J.V. (2013). Avian higher-level relationships and classification: Nonpasseriforms. The Howard and Moore Complete Checklist of the Birds of the World.

