# Supplementary material for: Parsimony and Model-Based Analyses of Indels in Avian Nuclear Genes Reveal Congruent and Incongruent Phylogenetic Signals
Source: Biology (Basel). 2013 Mar 13;2(1):419–44. doi: 10.3390/biology2010419 (PMC4009869; doi:10.3390/biology2010419)
Supplement: Supplementary File 1 — Supplementary Information (ZIP, 104 KB) [file biology-02-00419-s001.zip › biology-28592-supplementary-layout-final/supporting file 2.doc]

*Biology* **2013**, *2*, 1-x manuscripts; doi:10.3390/biology20x000x

**OPEN ACCESS**

***biology***

**ISSN 2079-7737**

www.mdpi.com/journal/biology

Article

Parsimony and Model-Based Analyses of Indels in Avian Nuclear Genes Reveal Congruent and Incongruent Phylogenetic Signals

Tamaki Yuri ^1,2^, Rebecca T. Kimball ^1^, John Harshman ^3^, Rauri C. K. Bowie ^4^,
Michael J. Braun ^5,6^, Jena L. Chojnowski ^1^, Kin-Lan Han ^1^, Shannon J. Hackett ^7^,
Christopher J. Huddleston ^5^, William S. Moore ^8^, Sushma Reddy ^9^, Frederick H. Sheldon ^10^, David W. Steadman ^11^, Christopher C. Witt ^12^ and Edward L. Braun ^1,^*

^1^	Department of Biology, University of Florida, Gainesville, FL 32611, USA;
E-Mails: tyuri@ou.edu (T.Y.); rkimball@ufl.edu (R.T.K.); kixs4@uga.edu (J.L.C.); hankin@ufl.edu (K.-L.H.)

^2^	Sam Noble Oklahoma Museum of Natural History, University of Oklahoma, Norman,
OK 73072, USA

^3^	4869 Pepperwood Way, San Jose, CA 95124, USA; E-Mail: jharshman@pacbell.net

^4^	Museum of Vertebrate Zoology and Department of Integrative Biology, University of California, Berkeley, CA 94720, USA; E-Mail: bowie@berkeley.edu

^5^	Department of Vertebrate Zoology, National Museum of Natural History, Smithsonian Institution, 4210 Silver Hill Road, Suitland, MD 20746, USA; E-Mails: braunm@si.edu (M.J.B.); huddlestonc@si.edu (C.J.H.)

^6^	Behavior, Ecology, Evolution, and Systematics Program, University of Maryland, College Park, MD 20742, USA

^7^	Zoology Department, Field Museum of Natural History, 1400 South Lakeshore Drive, Chicago,
IL 60605, USA; E-Mail: shackett@fieldmuseum.org

^8^	Department of Biological Sciences, Wayne State University, 5047 Gullen Mall, Detroit, MI 48202, USA; E-Mail: wmoore1415@gmail.com

^9^	Biology Department, Loyola University Chicago, Chicago, IL 60660, USA; E-Mail: sreddy6@luc.edu

^10^	Museum of Natural Science, 119 Foster Hall, Louisiana State University, Baton Rouge, LA 70803, USA; E-Mail: fsheld@lsu.edu

^11^	Florida Museum of Natural History, University of Florida, Gainesville, FL 32611, USA;
E-Mail: dws@flmnh.ufl.edu

^12^	Department of Biology and Museum of Southwestern Biology, University of New Mexico, Albuquerque, NM 87131, USA; E-Mail: cwitt@unm.edu

*****	Author to whom correspondence should be addressed; E-Mail: ebraun68@ufl.edu;
Tel.: +1-352-846-1124; Fax: +1-352-392-3704.

Received: 28 December 2012; in revised form: 21 February 2013 / Accepted: 22 February 2013 /
Published:

Appendix

1. Introduction

Here we consolidate and extend the current supra-ordinal taxonomy for birds to facilitate discussion of the performance of analyses using gap characters. We follow our description of nomenclature with a brief review of the support for those groups in our analyses of gap characters. This work should not be viewed as an effort to completely revise avian taxonomy. Nonetheless, we felt it was a useful exercise to summarize proposed clade names and propose additional names based upon Hackett *et al.* [1]. Given the emerging support for a robust avian phylogeny based upon analyses of nuclear sequence data [1–5] and rare genomic changes [6,7] a more complete taxonomic revision will be warranted soon. To facilitate such efforts we complete this appendix by discussing issues associated with the existing supra-ordinal clade names and by providing a brief comparison to the forthcoming *Howard and Moore Complete Checklist of the Birds of the World*.

2. Clade Names

2.1. Supra-Ordinal Clades Recovered in Multi-Gene Analyses

Throughout this section the letters used to identify clades refer to the labels in Figure 1 in the main text (the “Early Bird tree”). The named clades in the Early Bird tree that are recovered in analyses of gap characters are listed in Table 1 of this appendix (also see Figure 3 in the main text).

**Table 1.** Named supra-ordinal clades recovered in analyses of gap characters (numbers indicate bootstrap support; “—” indicates that the clade was not recovered).

| **Name** | **Independent Support?** | **ML** | **MP** |
| --- | --- | --- | --- |
| Psittacopasserae | + | — | <50% |
| Eufalconimorphae | + | — | — |
| Australaves | + | — | — |
| Picodynastornithes | + | 82% | 75% |
| Picocoraciae | + | 51% | <50% |
| Eucavitaves | + | — | — |
| Cavitaves |  | — | — |
| Telluraves | + | <50% | — |
| Litoritelluraves |  | — | — |
| Austrodyptornithes |  | — | <50% |
| Aequornithes | + | — | — |
| Insolitaves |  | — | — |
| Strisores |  | <50% | <50% |
| Novaeratitae | + | 95% | 94% |
| Notopalaeognathae | + | — | — |

Clade A: (Independently Corroborated)

Suh *et al.* [6] proposed the name **Psittacopasserae** for the clade comprising Passeriformes (perching birds) and Psittaciformes (parrots). This clade corresponds to Cantiomimus of Ericson [8] but we note that Psittacopasserae has informal priority.

Clade B: (Independently Corroborated)

Suh *et al.* [6] proposed the name **Eufalconimorphae** for the clade comprising Psittacopasserae and Falconiformes (falcons). This name presumably refers to the hypothesis that the ancestor of this clade was probably raptorial (see Mayr [9]), although Suh *et al.* [6] did not state this explicitly. Although this name has informal priority, we consider it problematic (see below, Section 3).

Clade C: (Independently Corroborated)

Ericson [8] proposed the name **Australaves** as the clade comprising Euaustralaves and Cariamiformes (seriemas) based upon a biogeographic hypothesis that places the centers of origin for these taxa on southern continents. Note that Ericson [8] originally proposed “Australavis” but we have modified the ending to avoid the singular “avis”. This clade corresponds to the “PPFC clade” of Wang *et al.* [3].

Clade D: (Independently Corroborated)

We propose the name **Picodynastornithes** for the clade comprising Coraciiformes (kingfishers, rollers, and allies) and Piciformes (woodpeckers and allies). Picodynastornithes is from the Latin *picus* (m., woodpecker) and the Greek *dynastes* (m., lord, master, ruler) in reference to the “king” in kingfishers (Alcedinidae *sensu lato*, corresponding to the circumscription used in the Clements checklist [10]).

Clade E: (Independently Corroborated)

Mayr [11] proposed the name **Picocoraciae** for the clade comprising Picodynastornithes and Bucerotiformes (hornbills, hoopoes, and woodhoopoes).

Clade F: (Independently Corroborated)

We propose the name **Eucavitaves** for the clade comprising Picocoraciae and Trogoniformes (trogons). **Eucavitaves** is derived from the Latin *cavus* (hollow, hole), refering to the cavity-nesting habits of the clade, and *aves* (birds) combined with the Greek *eu* (well, good) to indicate that this group corresponds to the core Cavitaves (see below). This clade corresponds to the “CPBT clade” of Wang *et al.* [3].

Clade G:

We propose the name **Cavitaves** for the clade comprising Eucavitaves and Leptosomiformes (the monotypic order for the cuckoo roller *Leptosomus discolor*). Like Eucavitaves, it is derived from the Latin *cavus* (hollow, hole) and *aves* (birds).

Clade H: (Independently Corroborated)

We propose the name **Telluraves** for the “landbird” clade of Hackett *et al.* [1], which comprises Falconimorphae and Cavitaves as well as the Accipitriformes (hawks and New World vultures), Strigiformes (owls), and Coliiformes (mousebirds). **Telluraves** is derived from the Latin words *telluris* (f., the earth, earth, land) and *aves* (birds).

Clade I:

We propose the name **Litoritelluraves** for the clade comprising Telluraves and Charadriiformes (shorebirds). **Litoritelluraves** is derived from the Latin *litus, litoris* (n., shore), referring to shorebirds, and Telluraves.

Clade J:

We propose the name **Austrodyptornithes** for as the clade comprising Sphenisciformes (penguins) and Procellariiformes (petrels, shearwaters, and albatrosses). **Austrodyptornithes** is derived from the Greek word *dypto* (plunge in, dive) and the Latin *austri* (south) in reference to most of the species in this clade living in the southern hemisphere and diving into the ocean.

Clade K: (Independently Corroborated)

Mayr [11] proposed the name **Aequornithes** as the clade comprising Austrodyptornithes along
with Gaviiformes (loons), Ciconiiformes (storks), Suliformes (cormorants and allies), and Pelecaniformes (pelecans, herons, ibis, and allies, corresponding to the circumscription used in the Clements checklist [10]).

Clade L:

We propose the name **Insolitaves** for the clade comprising Aequornithes along with Cuculiformes (cuckoos), Gruiformes (*sensu stricto*, comprising only rails, cranes, and their close relatives), Musophagiformes (turacos), and Otidiformes (bustards). **Insolitaves** is derived from the Latin *insolitus* (unfamiliar, strange) and *aves* (birds), because the clade covers an extremely wide range of morphologies and habitats.

Clade M: (Independently Corroborated)

Mayr [12] revived the use of **Strisores** [13,14] for the clade comprising Apodiformes (swifts and hummingbirds) and traditional “Caprimulgiformes”. Traditional “Caprimulgiformes” corresponds to the *sensu lato* definition used by the Clements checklist [10], comprising the orders Aegotheliformes (owlet-nightjars), Caprimulgiformes (*sensu stricto*, nightjars and nighthawks), Podargiformes (frogmouths), Nyctibiiformes (potoos), and Steatornithiformes (the oilbird).

Clade Q: (Independently Corroborated)

We propose the name **Novaeratitae** for the clade comprising the Casuariiformes (cassowarys and emus) and Aptyrigiformes (kiwis), which are the extant Australasian ratites. Novaeratitae is from the Latin words *novus* (new) and *ratis* (f., a raft). *Novae* refers to the three regions where this clade is found, namely New Guinea (*novaeguineae*), Australia (*novaehollandiae*), and New Zealand (*novaeseelandiae*) and *ratis* refers to the unkeeled sternum of these birds.

Clade R: (Independently Corroborated)

We propose the name **Notopalaeognathae** for the clade comprising Novaeratitae, Tinamiformes (tinamous), and Rheiformes (rheas). Notopalaeognathae is from the Greek *notos* (southern) and Palaeognathae from the Greek word *palaios* (ancient, old) and *gnathidion* (jaw), the latter two words referring to the birds classified by Pycraft [15] as having a “primitive” (palaeognathous) palate. *Noto* refers to the distribution of these taxa on fragments of the ancient southern supercontinent Gondwana. It also refers to the exclusion of Struthioniformes (ostriches) from this clade since they historically had a widespread Eurasian distribution.

2.2. Supra-Ordinal Clades that Are Typically Recovered in Single Gene Analyses

In addition to the relatively difficult to recover supra-ordinal clades described above (and indicated with letters in Figure 1 in the main text), there are six very strongly supported supra-ordinal clades that are typically recovered in analyses of individual genes. These clades were omitted from Table 1 in the main text because they do not represent a rigorous test of the phylogenetic method we used (they are indicated with red asterisks in Figure 1 in the main text).

Four of these clades correspond to the major divisions in the avian tree of life. These clades have received extensive independent corroboration (reviewed by Cracraft *et al.* [16]). **Palaeognathae** [15] is the clade comprising Notopalaeognathae and Struthioniformes (ostriches). **Galloanserae** [17] is the clade comprising Galliformes (pheasants, partridges, and allies) and Anseriformes (ducks, geese, and allies). **Neoaves** [17] corresponds to the most species-rich Neornithine clade, comprising all extant birds with the exception of those in Galloanserae and Palaeognathae. Finally, **Neognathae** [15]
is the clade comprising Neoaves and Galloanserae (*i.e.*, all extant birds except the Palaeognathae). Sangster [18,19] introduced names for two additional clades: **Daedalornithes** (the clade comprising Aegotheliformes [owlet-nightjars] and Apodiformes [swifts and hummingbirds]) and **Mirandornithes** (the clade comprising Podicipediformes [grebes] and Phoenicopteriformes [flamingos]). All of these clades were recovered in analyses using only gap characters with bootstrap support ≥80%.

3. Notes on Terminology

3.1. Comments on Eufalconimorphae, Strisores, and Galloanserae

Several names for supra-ordinal clades in the avian tree of life deserve additional comment. First, we note that the use of Eufalconimorphae [6] shares an obvious connection to “Falconimorphae”, a name that has been used for the non-monophyletic grouping of raptors (e.g., Livezey and Zusi [20]). This might make it reasonable to revise “Falconimorphae” to correspond to Austalaves [8]. The prefix “eu-” has often been used for species-rich groups within a larger clade (*i.e.*, Eupasseres [21]; Eucavitaves in this manuscript), making that name a logical extension of the terminology proposed by Suh *et al.* [6]. However, given the potential for confusion due to the prior use of Falconimorphae for the non-monophyletic “raptorial” group, it seems inappropriate to use that name; instead we recommend continued use of Australaves.

Excluding Cariamiformes from Eufalconimorphae raises another concern. Ericson [8] pointed out that Cariamiformes are the ecological equivalent of the African secretarybird, *Sagittarius serpentarius*, which is a member of Accipitriformes (hawks and allies, including New World vultures). Thus, the name Eufalconimorphae places many non-raptorial taxa (Passeriformes and Psittaciformes) in the “true falcons” and excludes a raptorial taxon (Cariamiformes). We also note that Ericson [8] suggested subsuming Cariamiformes into Falconiformes, reflecting the recovery of a Falconiformes-Cariamiformes clade in that study. The relatively large number of TE insertions that support Eufalconimorphae [6] leads us to conclude that Falconiformes+Cariamiformes are paraphyletic, contrary to Ericson [8] but consistent with the fossil evidence that suggests the ancestor state for these taxa was raptorial [9]. Nonetheless, we note that Wang *et al.* [3] also recovered this Falconiformes-Cariamiformes clade, albeit with limited support. For this reason, we suggest that additional study of the potential for biased estimation of phylogeny in this part of the avian tree of life is desirable regardless of nomenclatural issues.

Second, priority for the name Strisores as used by Mayr [12] is also problematic. Mayr [22] revived the name Cypselomorphae, originally used by Huxley [23] for Apodiformes and Caprimulgiformes, using the name for the clade comprising Apodiformes, Caprimulgiformes (*sensu stricto*), and Nyctibiiformes. However, the least inclusive clade that comprises those taxa is identical to Strisores (*sensu* Mayr [12]) given the Early Bird tree. Analyses of gap characters (Figure 3 in the main text) support a third topology that is also consistent with maintaining a distinction between Strisores and Cypselomorphae. Despite this uncertainty, continued use of Strisores seems warranted. Use of the name Strisores for these taxa preceded the use of Cypselomorphae and it appears to be becoming more popular in modern literature (e.g., Nesbitt *et al.* [24]). Moreover, it is unlikely that the deepest divergence in Strisores was between Apodiformes and Caprimulgiformes (none of the estimates of phylogeny cited above are consistent with that topology). Thus, using Strisores for the larger clade and limiting Cypselomorphae to the clade comprising Apodiformes and Caprimulgiformes (*sensu stricto*), which is consistent its original definition [23] would resolve this confusion.

Strisores is also a problematic taxon from the standpoint of independent evidence. The criterion that we have used to establish whether clades have independent corroboration is a combination of analyses using data that are independent of Hackett *et al.* [1] along sufficient taxon sampling to provide a rigorous test of the clade of interest. No such test of Strisores has been published, although some recent publications based upon data independent of Hackett *et al.* [1] do provide support for monophyly for subsets of the taxa comprising Strisores (e.g., Wang *et al.* [3] and McCormack *et al.* [5]). Moreover, there is sufficient evidence from morphology (e.g., Livezey and Zusi [20]) to view Strisores as having a reasonable degree of independent corroboration. Analyses of gap characters alone (Figure 3 in the main text) also recover Strisores, albeit with <50% bootstrap support.

Finally, we note that Mayr [11] suggested that Galloanseres is preferable to Galloanserae. Sibley  *et al.* [17] proposed the name with the ending “-ae” based upon their DNA hybridization result that suggested that Galloanserae and Palaeognathae are sister taxa. This in turn suggested the division of extant birds into Palaeognathae, Galloanserae, and Neognathae. Subsequent analyses of many different types of data (reviewed by Cracraft *et al.* [16] and Mayr [11]) have confirmed the sister group relationship between Galloanseres and Neoaves, suggesting that the ending “-es” is to be preferred over “-ae”. However, we note that Galloanserae remains much more commonly used by the community. Moreover, the “-ae” ending has been maintained for some taxa included within Neoaves (e.g., Psittacopasserae and Eufalconimorphae). Thus, we have maintained the use of this name (e.g., supporting information, file 1).

3.2. Comparison to the Forthcoming Howard and Moore Taxonomy

Cracraft [25] presented a ranked classification of birds based upon the best current estimates of avian phylogeny as part of the forthcoming *Howard and Moore Complete Checklist of the Birds of the World*. Readers are referred to that work for details, but we have chosen to highlight a few differences from our proposed classification:

1. Telluraves and Aequornithes were assigned the rank of cohort. Cracraft [25] revived the use of Coracornithes [26] for Telluraves. In both cases, the ending was modified to “-ia” (*i.e.*, Coracornithia and Aequornithia).
2. Australaves, Austrodyptornithes, and Novaeratitae were assigned the rank of superorder. The names for these groups were Passerimorphae (=Australaves), Apterygimorphae (=Novaeratitae), and Procellariimorphae (=Austrodyptornithes).
3. Strisores [12] were subsumed into a single order (Caprimulgiformes) and the name Daedalornithes [18] was replaced by the Trochiloidea (which has the rank of superfamily).
4. Mirandornithes [19] were subsumed into a single order (Phoenicopteriformes). Grebes were reduced in rank to a family (Podicipedidae) within this order.

References and Notes

1.	Hackett, S.J.; Kimball, R.T.; Reddy, S.; Bowie, R.C.K.; Braun, E.L.; Braun, M.J.; Chojnowski, J.L.; Cox, W.A.; Han, K.-L.; Harshman, J.; *et al*. A phylogenomic study of birds reveals their evolutionary history. *Science* **2008**, *320*, 1763–1768.

2.	Ericson, P.G.P.; Anderson, C.L.; Britton, T.; Elzanowski, A.; Johansson, U.S.; Källersjö, M.; Ohlson, J.I.; Parsons, T.J.; Zuccon, D.; Mayr, G. Diversification of Neoaves: Integration of molecular sequence data and fossils. *Biol. Lett.* **2006**, *2*, 543–547.

3.	Wang, N.; Braun, E.L.; Kimball, R.T. Testing hypotheses about the sister group of the Passeriformes using an independent 30 locus dataset. *Mol. Biol. Evol.* **2012**, *29*, 737–750.

4.	Smith, J.V.; Braun, E.L.; Kimball, R.T. Ratite non-monophyly: Independent evidence from 40 novel loci. *Syst. Biol.* **2013**, *62*, 35–49.

5.	McCormack, J.E.; Harvey, M.G.; Faircloth, B.C.; Crawford, N.G.; Glenn, T.C.; Brumfield, R.T. A phylogeny of birds based on over 1,500 loci collected by target enrichment and high-throughput sequencing. *PLoS One* **2013**, *8*, e54848.

6.	Suh, A.; Paus, M.; Kiefmann, M.; Churakov, G.; Franziska, A.F.; Brosius, J.; Kriegs, J.O.; Schmitz, J. Mesozoic retroposons reveal parrots as the closest living relatives of passerine birds. *Nat. Commun.* **2011**, *2*, 443.

7.	Matzke, A.; Churakov, G.; Berkes, P.; Arms, E.M.; Kelsey, D.; Brosius, J.; Kriegs, J.O.;
Schmitz, J. Retroposon insertion patterns of neoavian birds: Strong evidence for an extensive incomplete lineage sorting era. *Mol. Biol. Evol.* **2012**, *29*, 1497–1501.

8.	Ericson, P.G.P. Evolution of terrestrial birds in three continents: Biogeography and parallel radiations. *J. Biogeogr.* **2012**, *39*, 813–824.

9.	Mayr, G. Well-preserved new skeleton of the Middle Eocene Messelastur substantiates sister group relationship between Messelasturidae and Halcyornithidae (Aves, ?Pan-Psittaciformes).
*J. Syst. Paleontol.* **2011**, *9*, 159–171.

10.	Clements, J.F.; Schulenberg, T.S.; Iliff, M.J.; Sullivan, B.L.; Wood, C.L.; Roberson, D.
The Clements Checklist of Birds of the World: Version 6.6. Available online: http://www.birds.cornell.edu/clementschecklist/downloadable-clements-checklist/ (accessed on 12 August 2012).

11.	Mayr, G. Metaves, Mirandornithes, Strisores and other novelties—A critical review of the
higher-level phylogeny of neornithine birds. *J. Zool. Syst. Evol. Res.* **2011**, *49*, 58–76.

12.	Mayr, G. Phylogenetic relationships of the paraphyletic “caprimulgiform” birds (nightjars and allies). *J. Zool. Syst. Evol. Res.* **2010**, *48*, 126–137.

13.	Cabanis, J.L. Ornithologische Notizen. *Arch. Naturgesch.* **1847**, *13*, 186–256, 308–352.

14.	Baird, S.F. *Reports of Explorations and Surveys: To Ascertain the Most Practicable and Economical Route for a Railroad from the Mississippi River to the Pacific Ocean*; Beverly Tucker: Washington, DC, USA, 1858; p. 1005.

15.	Pycraft, W.P. Part II. On the morphology and phylogeny of the Palaeognathae (Ratitae and Crypturi) and Neognathae (Carinatae). *Trans. Zool. Soc. Lond.* **1900**, *15*, 149–290.

16.	Cracraft, J.; Barker, F.K.; Braun, M.; Harshman, J.; Dyke, G.J.; Feinstein, J.; Stanley, S.;
Cibois, A.; Schikler, P.; Beresford, P.; *et al*. Phylogenetic Relationships among Modern Birds (Neornithes): Towards an Avian Tree of Life. In *Assembling the Tree of Life*; Cracraft, J., Donoghue, M., Eds.; Oxford University Press: Oxford, UK, 2004; pp. 468–489.

17.	Sibley, C.G.; Ahlquist, J.E.; Monroe, B.L. A classification of the living birds of the world based on DNA-DNA hybridization studies. *Auk* **1988**, *105*, 409–423.

18.	Sangster, G. A name for the clade formed by owlet-nightjars, swifts and hummingbirds (Aves). *Zootaxa* **2005**, *799*, 1–6.

19.	Sangster, G. A name for the flamingo-grebe clade. *Ibis* **2005**, *147*, 612–615.

20.	Livezey, B.C.; Zusi, R.L. Higher-order phylogeny of modern birds (Theropoda, Aves: Neornithes) based on comparative anatomy: II.—Analysis and discussion. *Zool. J. Linn. Soc.* **2007**, *149*, 1–94.

21.	Ericson, P.G.P.; Irestedt, M.; Johansson, U.S. Evolution, biogeography, and patterns of diversification in passerine birds. *J. Avian Biol.* **2003**, *34*, 3–15.

22.	Mayr, G. Osteological evidence for paraphyly of the avian order Caprimulgiformes (nightjars and allies). *J. Ornithol.* **2002**, *143*, 82–97.

23.	Huxley, T.H. On the classification of birds; and on the taxonomic value of the modifications of certain of the cranial bones observable in that class. *Proc. Zool. Soc. Lond.* **1867**, *145*, 415–472.

24.	Nesbitt, S.J.; Ksepka, D.T.; Clarke, J.A. Podargiform affinities of the enigmatic *Fluvioviridavis platyrhamphus* and the early diversification of Strisores (“Caprimulgiformes” + Apodiformes). *PLoS One* **2011**, *6*, e26350.

25.	Cracraft, J. Avian higher-level relationships and classification: Nonpasseriforms. In *The Howard and Moore Complete Checklist of the Birds of the World, 4th edition. Volume 1: Non-passerines*; Dickinson, E.C., Remsen, J.V., Eds.; Aves Press: Northhampton, UK, in press.

26.	Fürbringer, M. *Untersuchungen zur Morphologie und Systematik der Vögel, zugleich ein Beitrag zur Anatomie der Stütz- und Bewegungsorgane*; T. J. van Holkema: Amsterdam, The Netherlands, 1888.

© 2013 by the authors; licensee MDPI, Basel, Switzerland. This supporting information is an open access document distributed under the terms and conditions of the Creative Commons Attribution license (http://creativecommons.org/licenses/by/3.0/).
